# Real-World Outcomes of Antifungal Prophylaxis in Adult Acute Lymphoblastic Leukemia Patients: A Multicenter Comparison of the Use of Fluconazole and Micafungin

**DOI:** 10.3390/jcm14207294

**Published:** 2025-10-16

**Authors:** Unal Atas, Utku Iltar, Orhan Kemal Yucel, Hasan Salur, Merve Cagla Bilek, Tayfun Ustabas, Ozlem Candan, Gulten Korkmaz, Selin Kucukyurt, Pinar Tiglioglu, Sureyya Yigit Kaya, Burak Deveci, Atakan Tekinalp, Rafiye Ciftciler, Aysun Senturk Yikilmaz, Tayfun Elibol, Tayfur Toptas, Ahmet Kursad Gunes, Omur Gokmen Sevindik, Leylagul Kaynar, Rabin Saba, Isik Atagunduz, Gulsum Ozet, Volkan Karakus, Ozan Salim, Erdal Kurtoglu, Levent Undar

**Affiliations:** 1 Faculty of Medicine, Department of Internal Medicine, Division of Hematology, Akdeniz University, Antalya 07100, Turkey; vrlunalatas@gmail.com (U.A.); okyucel@hotmail.com (O.K.Y.); mccagla06@gmail.com (M.C.B.); ozansalim@gmail.com (O.S.); leventundar@gmail.com (L.U.); 2 Department of Internal Medicine, Antalya City Hospital, Antalya 07080, Turkey; hasansalur@gmail.com; 3 Division of Hematology, Health Sciences University, Antalya Training and Research Hospital, Antalya 07100, Turkey; tayfunustabas1@gmail.com (T.U.); dr_v_karakus@yahoo.com (V.K.); erdalkurtoglu@yahoo.com (E.K.); 4 Faculty of Medicine, Division of Hematology, Marmara University, Istanbul 34854, Turkey; ozlemego@gmail.com (O.C.); toptast@gmail.com (T.T.); isikkaygusuz@yahoo.com (I.A.); 5 Division of Hematology, Ankara Bilkent City Hospital, Ankara 06800, Turkey; drgulten@gmail.com (G.K.); gulsumozet@gmail.com (G.O.); 6 Division of Hematology, Ankara Etlik City Hospital, Ankara 06170, Turkey; dr.skucukyurt@hotmail.com (S.K.); ahmetkgunes@gmail.com (A.K.G.); 7 Faculty of Medicine, Division of Hematology, Istanbul Medeniyet University, Istanbul 34700, Turkey; dr.pinarakyol@hotmail.com; 8 Faculty of Medicine, Division of Hematology, Istanbul Medipol University, Istanbul 34815, Turkey; sureyyayigitkaya@hotmail.com (S.Y.K.); omurgok17@hotmail.com (O.G.S.); drleylagul@gmail.com (L.K.); 9 Faculty of Medicine, Division of Hematology, Antalya Bilim University, Antalya 07190, Turkey; deveci.burak@gmail.com (B.D.); rabinsaba@gmail.com (R.S.); 10 Faculty of Medicine, Division of Hematology, Necmettin Erbakan University, Konya 42090, Turkey; atakantekinalp@hotmail.com; 11 Faculty of Medicine, Division of Hematology, Selcuk University, Konya 42250, Turkey; rafiyesarigul@gmail.com; 12 Division of Hematology, Denizli State Hospital, Denizli 20010, Turkey; senturkaysun@gmail.com; 13 Division of Hematology, Göztepe Prof. Dr. Suleyman Yalcin City Hospital, Istanbul 34722, Turkey; tayfunelibol@gmail.com

**Keywords:** acute lymphoblastic leukemia, antifungal prophylaxis, fluconazole, micafungin, invasive fungal infection

## Abstract

**Background:** Adult acute lymphoblastic leukemia (ALL) patients are at increased risk of invasive fungal infections (IFIs) due to intensive therapy and prolonged neutropenia. While pediatric guidelines support administering fluconazole or mold-active agents, the evidence in adults is limited. This study presents the first multicenter retrospective comparison of fluconazole and micafungin use in this setting. **Methods:** We retrospectively analyzed 336 adult ALL patients from 11 centers in Türkiye (2010–2024) who received fluconazole (*n* = 230) or micafungin (*n* = 106) during induction chemotherapy. IFIs were classified according to the EORTC/MSG criteria. **Results:** The median age was 38.5 years, and 38.7% were female. Proven/probable IFIs occurred in 8.9% of patients, with similar rates between the fluconazole and micafungin groups (8.7% vs. 9.4%; *p* = 0.82). Multivariate analysis confirmed no significant association between the prophylactic antifungal type and IFI incidence, indicating comparable outcomes across groups. The median prophylaxis duration was longer with fluconazole, while the discontinuation rates, switch patterns, and subsequent antifungal use were comparable. The overall infection rates (~60%) and distribution of bacterial, viral, and polymicrobial infections were similar between the two groups. Prior bacterial infection increased the risk of IFI by 2.7-fold, and IFI-positive patients had longer neutropenia. At the end of induction, the remission, refractory, and mortality rates were similar between groups. The median overall survival was 24 months. **Conclusions:** Fluconazole and micafungin showed similar efficacy as the primary antifungal prophylaxis treatment in adult ALL patients. Given the limited evidence in adults and the ongoing need to optimize antifungal strategies, prospective randomized trials directly comparing these agents in this population are needed to confirm and expand upon our findings.

## 1. Introduction

Invasive fungal infections (IFIs) are a major cause of morbidity and mortality in patients with acute leukemia and in those undergoing allogeneic hematopoietic stem cell transplantation (HSCT). Despite newer antifungal agents such as triazoles and echinocandins, IFIs remain a significant problem in hematologic malignancies. In a pediatric acute lymphoblastic leukemia (ALL) study, the 5-year cumulative incidence of infection-related mortality was 2.4%, accounting for 30% of all deaths; among infection-related deaths, 68% were bacterial, 20% were fungal, and 12% were viral [1]. In ALL patients, the reported incidence of IFIs ranges from 4% to 18%, comparable to rates observed in acute myeloid leukemia (AML) patients [2,3,4,5,6]. Despite methodological differences and heterogeneous patient populations, the reported incidence of IFIs in pediatric and adult ALL patients appears to be within a similar range [3,4,5,6]. Pediatric guidelines recommend primary antifungal prophylaxis in high-risk patients, with fluconazole advised in centers with a low incidence of invasive mold infections due to its efficacy against *Candida* species [7]. In contrast, evidence supporting prophylaxis in adult ALL patients remains limited and heterogeneous. Prophylaxis is generally not recommended for those receiving tyrosine kinase inhibitors (TKIs) therapy alone, although fluconazole may be cautiously considered in standard-risk patients to prevent *Candida* infections. In high-risk patients, particularly those with prolonged neutropenia following intensive chemotherapy, mold-active agents such as liposomal amphotericin B or echinocandins may be considered; however, there is no definitive evidence to support their clear clinical benefit [2,7].

In ALL patients, the heightened risk of *Candida* infections, together with the increased susceptibility to mold infections in high-risk subgroups, underscores the need to determine the optimal choice of antifungal prophylaxis [5,7]. Echinocandins are active against most Candida species—including *C. krusei* and *C. glabrata*, which are frequently resistant to fluconazole—and exert fungicidal activity against *Pneumocystis jirovecii* and fungistatic effects against Aspergillus species [3,8]. Prospective and retrospective studies comparing mold-active agents (e.g., voriconazole, micafungin, caspofungin) with non-mold-active agents (e.g., fluconazole) have been conducted primarily in pediatric patients undergoing HSCT or diagnosed with AML [2,7]. A meta-analysis of these studies suggested that mold-active prophylaxis was more effective than fluconazole in preventing IFIs, but without a significant impact on overall survival (OS). Additionally, micafungin offers the advantage of fewer drug–drug interactions; however, its higher cost remains a notable drawback [9]. Although these pharmacological features make micafungin appealing for prophylaxis, evidence for its use in adult ALL patients remains limited, and most data are extrapolated from pediatric cohorts, AML populations, or HSCT settings [2,3,7,8,9].

Consequently, the optimal antifungal prophylaxis strategy for adult ALL patients during induction therapy remains unclear, and the practice patterns vary widely across institutions. While fluconazole has been the traditional choice due to its activity against *Candida*, micafungin offers broader coverage and fewer drug–drug interactions; however, its routine use remains unsupported by strong evidence. Real-world evidence directly comparing fluconazole with micafungin in adult ALL patients is lacking, particularly across diverse treatment centers. Addressing this knowledge gap is critical for guiding future guidelines, harmonizing institutional practices, and improving outcomes in this high-risk population.

Here, we present the first multicenter retrospective study directly comparing fluconazole and micafungin as the primary antifungal prophylaxis during induction therapy in adult ALL patients. This study also evaluates the overall infection incidence, treatment outcomes, and survival, providing real-world data that may inform future prospective studies and help optimize prophylaxis strategies in adult ALL patients.

## 2. Materials and Methods

### 2.1. Study Design and Patient Population

Between January 2010 and December 2024, archival records of patients diagnosed with ALL at 11 centers across Türkiye were retrospectively reviewed. Adult patients (aged ≥18 years) with a confirmed diagnosis of ALL who had received at least one cycle of intensive induction chemotherapy and were administered either fluconazole or micafungin as the primary antifungal prophylaxis were included in the study. Patients were excluded if they did not receive antifungal prophylaxis, received agents other than fluconazole or micafungin, or had incomplete or missing clinical records. A total of 351 patients were screened, and 15 were excluded due to no antifungal prophylaxis, non-induction therapy, loss to follow-up during induction, or incomplete data, resulting in 336 patients included in the analysis. The study was approved by a local ethics committee.

For each patient, the following data were collected: age, sex, comorbidities, performance status, date of diagnosis, ALL subtype, Philadelphia (Ph) chromosome status (and the TKIs used if Ph-positive), induction regimen, the antifungal agent used for prophylaxis, baseline leukocyte and neutrophil counts, duration of neutropenia, occurrence of fungal infection during induction, duration and discontinuation status of antifungal prophylaxis, post-induction remission status, receipt of consolidation therapy, autologous HSCT status, disease status at last follow-up, survival status, and OS time. Neutropenia was defined as the absolute neutrophil count (ANC) < 1 × 10^9^/L and its duration as the cumulative number of days below this level during follow-up. OS was defined as the time from diagnosis to last follow-up or death.

### 2.2. Definitions of Invasive Fungal Infections

IFIs were classified according to the criteria established by the European Organization for Research and Treatment of Cancer/Mycoses Study Group (EORTC/MSG), which are widely adopted in clinical studies to standardize definitions among immunocompromised populations such as those in hematology–oncology cohorts. Based on these criteria, IFIs are categorized as proven, probable, or possible, depending on the combination of host factors, clinical features, and mycological evidence. Proven IFI requires histopathologic or microbiological confirmation; probable IFI includes the presence of host risk factors, consistent clinical signs, and mycological findings; and possible IFI includes compatible clinical findings and host factors in the absence of mycological evidence. In accordance with conventional methodology in prospective or randomized trials, only proven and probable cases were considered IFI-positive in this study [10].

To ensure consistency and reliability across all participating centers, we collected not only the categorical IFI classification but also the underlying diagnostic data including microbiological cultures, serum Aspergillus galactomannan antigen results, and high-resolution thoracic computed tomography (HRCT). These data were re-evaluated centrally according to the EORTC/MSG criteria to confirm that each case met the respective definitions. This two-step verification process allowed uniform application of the criteria and minimized inter-center variability.

### 2.3. Study Objectives

The primary objective was to compare the incidence of IFIs and the rate of prophylaxis discontinuation or switching to another antifungal agent between patients who received fluconazole and those who received micafungin as primary prophylaxis. The secondary objective was to evaluate the current incidence and distribution of infections in ALL patients, assess the clinical responses based on recent treatment approaches, and determine the OS outcomes.

### 2.4. Statistical Analysis

Statistical analyses were performed using SPSS version 25.0 (IBM Corp., Armonk, NY, USA). The distribution of continuous variables was assessed with the Shapiro–Wilk test. Cases with missing data for specific variables were excluded from that analysis (listwise deletion); no imputation was performed. Normally distributed variables were expressed as the mean ± standard deviation and non-normally distributed variables as the median (min–max). Categorical variables were reported as frequencies and percentages. Group comparisons were conducted using the independent samples *t*-test for normally distributed continuous variables and the Mann–Whitney U test for non-normal distributions. Categorical variables were analyzed using Pearson’s Chi-square test, Fisher’s exact test, likelihood ratio, or linear-by-linear association where appropriate. For multiple comparisons, the Benjamini–Hochberg false discovery rate (FDR) correction was applied.

OS was estimated using the Kaplan–Meier method, and differences between groups were compared using the log-rank test. A *p*-value < 0.05 was considered statistically significant.

## 3. Results

### 3.1. Patient Demographics, Clinical Features, and Treatment Characteristics

A total of 336 patients were included (351 initially screened; 15 excluded due to no prophylaxis, non-induction therapy, loss to follow-up, or incomplete data), with a median age of 38.5 years (range, 18–86), and 130 (38.7%) were female. At least one comorbidity was present in 62 patients (18.5%), most commonly hypertension (10.1%) and diabetes mellitus (7.7%). ECOG performance status was available for 279 patients, of whom 241 (71.7%) had a score of 0–1. The median duration of neutropenia during induction was 16 days (range, 0–55), with 297 patients (88.4%) experiencing neutropenia ≥ 10 days.

Overall, 80% of patients had B-cell ALL, and the HyperCVAD regimen was the most commonly used treatment protocol (225 patients; 66.9%). Eighty-six patients (25.6%) had Philadelphia chromosome-positive ALL, and all received TKI-based therapy during induction (imatinib in 57 [16.7%] and dasatinib in 31 [9.2%]).

Primary antifungal prophylaxis consisted of fluconazole in 230 patients (68.4%) and micafungin in 106 patients (31.6%). Compared with fluconazole, patients in the micafungin group were older (median 40 vs. 36 years, *p* = 0.016) and more frequently had ECOG 0–1 performance status (97.4% vs. 82.2%, *p* < 0.001). The treatment distribution also differed, with HyperCVAD being used more frequently in the micafungin group (77.4% vs. 62.2%), while adult asparaginase-containing protocols were more common in the fluconazole group (14.8% vs. 3.8%; *p* = 0.003). Details of the baseline demographic, clinical, laboratory, and induction treatment characteristics are presented in Table 1.

### 3.2. Antifungal Prophylaxis and Infectious Complications

The median duration of prophylactic antifungal use in the cohort was 24 days (range, 5–110). No IFI was observed in 286 patients (85.1%), possible IFI occurred in 20 patients (5.9%), and 30 patients (8.9%) developed IFI (23 probable, 7 proven). HRCT revealed findings compatible with fungal infection in 28 patients (8.3%), and serum Aspergillus antigen was positive in 7 patients (2.1%). Prophylaxis was discontinued in 51 patients (15.2%), with amphotericin B (51.0%) and voriconazole (37.3%) being the most frequent alternatives. The antifungal characteristics and IFI status are summarized in Table 2.

Non-fungal infections occurred in 208 patients (61.9%). Bacterial infections were the most common (30.9% of all patients), while viral infections were rare (1.8%). Mixed infections occurred in 17 patients (5.0%). Infection sites were documented in 113 patients, with the respiratory tract (11.9%), bloodstream/catheter (6.5%), and urinary tract (3.9%) as the leading foci. Microbiological confirmation was obtained in 39.4% of infection cases, most frequently *Escherichia coli* (22 patients), *Staphylococcus* spp. (16 patients), and *Klebsiella* spp. (13 patients). IFIs were microbiologically proven in seven patients (four *Candida*, three *Aspergillus*). Except for SARS-CoV-2 in five patients and respiratory syncytial virus in one patient, no other viral pathogens were confirmed.

### 3.3. Antifungal Prophylaxis and Infectious Characteristics by Group

The duration of prophylaxis was longer with fluconazole (25 vs. 21 days; *p* = 0.003), but the discontinuation rates and subsequent antifungal switches were comparable between groups. In both groups, when a change in antifungal therapy was needed, the most frequently chosen agents were amphotericin B and voriconazole. The incidence of proven/probable IFI was similar (8.7% vs. 9.4%; *p* = 0.82), and no significant difference was observed in the time to IFI development (Table 2).

With respect to non-fungal infections, the overall infection rate was similar between groups (~60%; *p* = 0.29). The distribution of bacterial, viral, fungal, and polymicrobial infections did not differ significantly (*p* = 0.95). However, subgroup analyses revealed some differences in infection patterns: respiratory tract infections were more frequent in the fluconazole group (48.6%), while urinary tract infections (15.0%) and multi-site infections (20.0%) were more common in the micafungin group (likelihood ratio test: χ^2^ = 15.668, df = 8, *p* = 0.047; Fisher’s Exact test: *p* = 0.026, Monte Carlo 99% CI: 0.022–0.030).

### 3.4. Predictors of Invasive Fungal Infection

The incidence of IFI did not differ by age (≥65 vs. <65 years, 10.7% vs. 8.9%; *p* = 0.44), ALL subtype, treatment regimen, or Ph chromosome status. The IFI rates across treatment groups were 10.7% in HyperCVAD, 8.2% in pediatric-inspired protocols, 2.6% in adult asparaginase-containing regimens, and 0% in low-intensity regimens (*p* = 0.272).

Patients with bacterial infection had a significantly higher incidence of IFI compared to those without infection (16.7% vs. 6.6%; *p* = 0.008). Similarly, IFI-positive patients had a significantly longer neutropenia duration than IFI-negative patients (23.6 ± 9.8 vs. 16.6 ± 7.6 days; *p* < 0.001). As the majority of patients had neutropenia lasting ≥10 days, the risk of IFI appeared similar between ≥10 and <10 days (9.4% vs. 5.6%, *p* = 0.44); however, ROC analysis identified 15 days as the optimal cut-off, with neutropenia ≥15 days conferring a 5.4-fold higher risk (AUC = 0.719; *p* < 0.001; OR = 5.36; 95% CI: 1.83–15.75). In contrast, the prophylactic antifungal type, duration of prophylaxis, and baseline leukocyte counts were not associated with IFI risk.

In univariate logistic regression, prolonged neutropenia duration and the presence of bacterial infection were significantly associated with IFI development, while age, sex, comorbidity, ECOG performance, ALL subtype, Ph positivity, and prophylactic antifungal type were not predictive (all *p* > 0.05). In the multivariate model, including the entire cohort, both prolonged neutropenia and bacterial infection were identified as independent predictors of IFI. Each additional day of neutropenia increased the risk of IFI by approximately 9% (OR = 1.09; 95% CI: 1.04–1.13; *p* < 0.001). Patients with concurrent bacterial infections had a 2.5-fold higher risk of IFI compared to those without (OR = 0.40; 95% CI: 0.18–0.88; *p* = 0.023, reverse-coded). The overall model was significant (χ^2^ = 22.3, *p* < 0.001), with a Nagelkerke R^2^ of 0.14, indicating moderate explanatory power (Table 3).

### 3.5. Treatment Responses, Disease Course, and Survival Outcomes

Of 318 evaluable patients, 265 (78.9%) achieved remission, 40 (11.9%) had refractory disease, and 13 (3.9%) died before response assessment, including 9 infection-related deaths (1 fungal). The response rates were comparable between the fluconazole and micafungin groups (remission: 83.6% vs. 82.7%; refractory: 11.8% vs. 14.3%; death: 4.5% vs. 3.1%; *p* = 0.70).

During the disease course, 126 patients (37.5%) developed relapse or refractory disease, and 157 (46.7%) underwent allogeneic HSCT. At last follow-up, 91 (27.1%) had active disease, and 181 (53.9%) had died, including 72 infection-related deaths (seven due to IFIs). The median OS was 24 months (range, 1–170).

Multivariate Cox regression identified age (HR = 1.035; 95% CI: 1.018–1.052; *p* < 0.001), relapse/refractory disease (HR = 0.492; 95% CI: 0.338–0.717; *p* < 0.001), and T-ALL subtype (HR = 0.597; 95% CI: 0.364–0.980; *p* = 0.041) as independent predictors of OS, while the prophylactic antifungal type was not associated with survival outcomes.

## 4. Discussion

This multicenter retrospective study is the first to directly compare fluconazole and micafungin as the primary antifungal prophylaxis during induction therapy in adult ALL patients. IFI occurred in 8.9% of patients, and the incidence was similar between the fluconazole and micafungin groups. The median prophylaxis duration was longer with fluconazole, but the discontinuation, switch rates, and subsequent antifungal use were similar. No associations were found with age, ALL subtype, Ph-positivity, or treatment protocol, whereas prior bacterial infection increased the IFI risk 2.7-fold, and IFI was linked to longer neutropenia.

ALL patients are at increased risk of IFI due to disease-related factors such as prolonged neutropenia, intensive polychemotherapy, and corticosteroid use [2,3,5]. Direct comparison of IFI risk between adult and pediatric ALL patients is not appropriate because of the differences in age, comorbidities, and genetic background [11]. Unlike pediatric ALL patients, where the IFI risk is considered low, data in adults are limited, and some studies report incidences similar to AML in the absence of mold-active prophylaxis [3,8,12]. The role of mold-active prophylaxis in ALL has not been well established, as evidence relies mainly on retrospective studies [11]. While fluconazole is widely used in children, its adequacy in adults is questioned; furthermore, although echinocandins provide extended activity, their high cost and lack of oral formulations restrict routine use [8].

The reported IFI incidence in pediatric and adult ALL patients ranges between 4 and 18% [2], which is consistent with our findings. Additionally, IFI incidence among adults treated with pediatric-inspired regimens was 8.2%, similar to that reported in pediatric cohorts [4,5,6]. In a large pediatric study of 6136 patients, the induction and consolidation phases, older childhood/adolescence, and poor initial response to therapy were identified as the highest-risk settings for IFI [5]. In adults, a study of 83 patients (83% receiving liposomal amphotericin B or posaconazole prophylaxis) showed no difference in IFI incidence between HyperCVAD (4%), BFM95 (15%), and Hoelzer (9%) protocols [13]. Similarly, in our cohort, the IFI rates were comparable across HyperCVAD (10.2%), adult asparaginase-containing regimens (2.6%), and pediatric-inspired regimens (8.2%). Previous studies reported that IFI was associated with prolonged and profound neutropenia, whereas a simple 10-day threshold was not predictive [5,6,12]. Similarly, in our cohort, the baseline neutrophil count and neutropenia ≥ 10 vs. < 10 days were not significantly different; indeed, as the majority of patients had neutropenia ≥ 10 days (*n* = 297, 88.4%), this cut-off lacked discriminatory power. In contrast, prolonged neutropenia was associated with higher risk, and ROC analysis identified 15 days as the optimal cut-off, with patients experiencing ≥ 15 days of neutropenia having a 5.4-fold higher incidence of IFI. Clinically, identifying a 15-day neutropenia threshold provides a practical marker to stratify IFI risk in adult ALL patients. The shift from the traditional 10-day threshold to 15 days likely reflects changes in treatment intensity and widespread prophylaxis use. Incorporating this updated threshold into future risk-stratification models may help tailor prophylaxis strategies and monitoring intensity. Consistent with earlier reports, most IFIs were mold-related and originated from the respiratory tract [4,6], and the incidence of IFI was also higher among patients who experienced bacterial infections.

Different antifungals have been evaluated for IFI prophylaxis across various hematologic malignancies, particularly in pediatric patients [14,15,16,17,18]. Two randomized controlled trials in pediatric and adult allogeneic and autologous HSCT recipients compared fluconazole with micafungin, showing higher prophylactic success with micafungin but no differences in proven/probable IFI, overall mortality, or fungal-related death; micafungin was also better tolerated in both studies [14,15]. A trial comparing micafungin with fosfluconazole, a phosphate prodrug of fluconazole, in pediatric malignancy and HSCT patients demonstrated comparable efficacy and safety [16]. Prophylactic amphotericin B did not provide superiority over the placebo [17]. Additionally, in an another pediatric acute leukemia study (68% ALL), low-dose amphotericin B was compared with oral voriconazole, and no difference was found in proven or probable IFI incidence [18]. A meta-analysis of 20 studies reported that mold-active antifungals, including echinocandins, reduced the risk of IFI and IFI-related mortality compared with fluconazole, but this benefit did not translate into improved OS, and the risk of adverse events leading to discontinuation or modification was higher with other azoles. However, all included studies were conducted in HSCT recipients and/or patients with various hematologic malignancies, and none focused exclusively on ALL [9].

In terms of adult ALL, no randomized trials have evaluated antifungal prophylaxis, and the available data are largely retrospective. In one study of 98 patients, 83 received primary prophylaxis during induction or consolidation, most commonly with liposomal amphotericin B (65%), posaconazole (18%), or fluconazole (8%). IFI occurred in 6% of prophylaxis recipients compared with 26.6% of those without prophylaxis [13]. In another retrospective study of 103 adult ALL patients, fluconazole and mold-active triazoles showed similar overall IFI incidence (17.6% vs. 15.9%), though mold-active triazoles tended to reduce invasive aspergillosis (11.8% vs. 1.4%; *p* = 0.07) [19]. In a study of 65 adult patients with acute leukemia, including 31 (47%) with ALL, IFI was reported in three patients (4.6%) under micafungin prophylaxis, one of whom had ALL [20]. In our study, which included a substantially larger cohort, the incidence of proven/probable IFI during induction was 9.4% among 106 adult ALL patients receiving micafungin prophylaxis and 8.9% overall across the fluconazole and micafungin groups (*n* = 336). Collectively, these findings indirectly suggest that fluconazole or micafungin may represent more suitable options for primary antifungal prophylaxis in adult ALL patients compared with amphotericin B or other triazoles, which carry higher risks of toxicity and drug interactions. Beyond efficacy, practical considerations influence antifungal selection in adult ALL. Fluconazole is inexpensive, widely available, and orally administered but lacks mold coverage and has significant CYP450-mediated drug interactions. Micafungin, while mold-active and generally better tolerated with fewer drug–drug interactions, is more costly and requires intravenous administration. Balancing these factors is essential for optimizing prophylaxis in different clinical settings.

In summary, the risk of mold-active IFI is lower in pediatric ALL patients compared with AML and allogeneic HSCT patients, and the predominance of *Candida* infections makes fluconazole a reasonable option. In adult ALL patients, however, a higher risk of mold-active IFI has been reported, although the available evidence remains limited. Overall, the data on antifungal prophylaxis in adult ALL are insufficient and inconsistent. In line with the literature, both the European Conference on Infections in Leukaemia (ECIL) and the Infectious Diseases Working Party (AGIHO) recommend antifungal prophylaxis for patients receiving chemotherapy, except for those treated with TKIs monotherapy. Due to toxicity and drug–drug interactions, triazoles (voriconazole, posaconazole) are not generally favored, while fluconazole is considered safer despite limited adult data. Micafungin may be considered an alternative based on limited evidence, whereas liposomal amphotericin B is not recommended for prophylaxis because of its suboptimal efficacy and toxicity concerns [2,21].

This study has several limitations. First, its retrospective design may have introduced selection and reporting bias, and some relevant clinical data could not be uniformly captured across all participating centers. Second, the treatment protocols were heterogeneous and evolved over the extended study period (2010–2024), reflecting changes in clinical practice, diagnostics, and antifungal prophylaxis strategies. This temporal variability may have influenced the results and limited our ability to fully control for potential confounders. Third, although the logistic regression model was statistically significant, its explanatory power was modest (Nagelkerke R^2^ = 0.14), and the sensitivity for identifying IFI-positive patients remained low (3.3%), indicating that additional unmeasured factors likely contributed to the IFI risk. Fourth, although proven cases allowed distinction between Candida and Aspergillus, species-level identification for probable or possible IFIs was limited, as these diagnoses relied mainly on imaging and clinical criteria rather than definitive microbiological confirmation. Finally, the results may not be generalizable to settings with different epidemiological profiles or institutional practices.

Future research should include prospective, randomized trials with standardized protocols to validate these findings in adult ALL and to compare mold-active agents with azoles. Such studies and multicenter registries could better define optimal prophylaxis strategies, identify high-risk subgroups, and inform national and international guidelines.

## 5. Conclusions

In this first multicenter analysis of adult ALL patients, fluconazole and micafungin showed comparable efficacy as the primary antifungal prophylaxis treatment. Prolonged neutropenia and bacterial infections, but not antifungal type, were identified as independent predictors of IFI. These findings support the continued use of fluconazole or micafungin in this setting, with agent selection best guided by local epidemiology, drug interactions, and cost considerations. Future prospective randomized trials with standardized protocols are needed to validate these retrospective findings and guide evidence-based antifungal prophylaxis strategies in adult ALL patients.

## Figures and Tables

**Table 1:** Demographic, laboratory, and treatment characteristics.

	All Patients (*n* = 336)	Fluconazole (*n* = 230)	Micafungin (*n* = 106)	*p* Value	*p* adj ^e^
**Age,** median (min–max), years	38.5 (18–86)	36 (18–85)	40 (18–86)	***0.016 ^1^***	*0.053*
**Sex,** *n* (%)				*0.812 ^2^*	*0.869*
Female	130 (38.7)	88 (38.2)	42(39.6)		
Male	206 (61.3)	142 (61.7)	64 (60.3)		
**Comorbidity,** *n* (%)	62 (18.5)	39 (16.9)	23 (21.7)	*0.298 ^2^*	*0.466*
Hypertension	34 (10.1)	22 (9.6)	12 (11.3)		
Diabetes mellitus	26 (7.7)	14 (6.1)	12 (11.3)		
Coronary artery disease	15 (4.5)	11 (4.8)	4 (3.8)		
Others ^a^	21 (6.3)	10 (4.3)	11 (10.4)		
**ECOG,** *n* (%)	279 (83.0)			***<0.001 ^2,3^***	***0.01***
0,1; *n* (%)	241 (71.7)	166 (82.2)	75 (97.4)		
2–4; *n* (%)	38 (11.3)	36 (17.8)	2 (2.6)		
**WBC at diagnosis,** median, ×10^9^/L (min–max)	15.0 (0.4–486.0)	18.46 (0.4–486.0)	12.24 (0.8–373.3)	*0.326 ^1^*	*0.466*
**ANC at diagnosis,** median, ×10^9^/L (min–max)	2.19 (0–106.0)	2.19 (0–85.0)	2.14 (0–106.0)	*0.869 ^1^*	*0.869*
**Neutropenia during induction,** median, days (min–max)	16 (0–55)	16 (0–55)	14 (5–42)	*0.078 ^1^*	*0.195*
**ALL subtype,** *n* (%)				*0.100 ^2^*	*0.200*
Pre B-ALL	119 (35.4)	89 (38.7)	30 (28.3)		
B-ALL	148 (44.0)	93 (40.5)	55 (51.9)		
T-ALL	56 (16.7)	40 (17.3)	16 (15.1)		
NA	13 (3.9)	8 (3.5)	5 (4.7)		
**Treatment protocols,** *n* (%)				***0.003 ^2^***	***0.015***
HyperCVAD	225 (66.9)	143 (62.2)	82 (77.4)		
Adult asparaginase-containing protocols ^b^	38 (11.3)	34 (14.8)	4 (3.8)		
Pediatric-inspired protocols ^c^	61 (18.2)	47 (20.4)	14 (13.2)		
Low-intensity protocols ^d^	12 (3.6)	6 (2.6)	6 (5.7)		
**Philadelphia chromosome positivity,** *n* (%)	86 (25.6)	60 (26.1)	26 (24.5)	*0.744 ^2^*	*0.869*
Imatinib	57 (16.7)	39 (17.0)	18 (17.0)		
Dasatinib	31 (9.2)	21 (9.1)	10 (9.4)		

ALL: acute lymphoblastic leukemia; ANC: absolute neutrophil count; ECOG: Eastern Cooperative Oncology Group; NA: not available; WBC: white blood cell. Continuous variables were expressed as the median (minimum–maximum), whereas categorical variables were expressed as *n* (%). ^a^ Chronic obstructive pulmonary disease; heart failure; hyperlipidemia; hypothyroidism; stroke; venous thrombosis. ^b^ In total, 28 patients received CALGB, 4 patients LINKER, 2 patients GMALL, and 4 patients other protocols. ^c^ In total, 26 patients received BFM, 18 patients DFCI, 10 patients GRAALL, and 8 patients NOPHO protocols. ^d^ Low-intensity therapy: Vincristine + steroid ± methotrexate and/or mercaptopurine. ^e^ Benjamini–Hochberg (FDR) correction was applied for multiple comparisons. *^1^ Mann–Whitney U test, ^2^ Pearson’s Chi-square test, ^3^ Fisher’s exact test.*

**Table 2:** Characteristics of antifungal prophylaxis and incidence of IFIs during induction.

	All Patients (*n* = 336)	Fluconazole (*n* = 230)	Micafungin (*n* = 106)	*p* Value
**Duration of antifungal prophylaxis,** median (min–max), days	24 (5–110)	25 (5–110)	21 (10–60)	***0.003 ^1^***
**Prophylaxis discontinued,** *n* (%)	51 (15.2)	34 (14.8)	17 (16.0)	*0.740 ^2^*
Switched antifungal agent *, *n* (%)				*0.470 ^3^*
Amphotericin B	26 (7.7)	16 (7.0)	10 (9.4)	
Voriconazole	19 (5.6)	12 (5.2)	7 (6.6)	
Posaconazole	2 (0.6)	2 (0.9)	0 (0)	
Other echinocandins	4 (1.2)	4 (1.7)	0 (0)	
**IFI status,** *n* (%)				*0.671 ^3^*
No IFI	286 (85.1)	197 (85.7)	89 (84.0)	
Possible IFI	20 (5.9)	13 (5.7)	7 (6.6)	
Probable IFI	23 (6.9)	14 (6.1)	9 (8.5)	
Proven IFI	7 (2.1)	6 (2.6)	1 (0.9)	
**IFI-positive patients** (probable or proven), *n* (%)	30 (8.9)	20 (8.7)	10 (9.4)	*0.820 ^2^*

IFI: invasive fungal infection. ^1^ *Mann–Whitney U test*, **^2^***Pearson’s Chi-square test*, **^3^***Fisher’s exact test.* * Among patients who discontinued prophylaxis (*n* = 51), of the switches, amphotericin B accounted for 51.0%, voriconazole 37.3%, posaconazole 3.9%, and other echinocandins 7.8%.

**Table 3:** Univariate and multivariate logistic regression analysis of factors associated with IFI development.

Variable	Univariate OR (95% CI)	*p* Value	Multivariate OR (95% CI)	*p* Value
**Sex** (Female vs. Male)	1.43 (0.68–3.05)	0.349	–	–
**Age at diagnosis**	1.01 (0.99–1.03)	0.370	–	–
**Philadelphia chromosome** (Ph+ vs. Ph-)	0.87 (0.36–2.11)	0.759	–	–
**Comorbidity** (Yes vs. No)	0.90 (0.35–2.30)	0.819	–	–
**White blood cell counts at diagnosis**	1.00 (1.00–1.00)	0.301	–	–
**Prophylactic antifungal** (Fluconazole vs. Micafungin)	0.91 (0.41–2.03)	0.825	–	–
**Duration of induction neutropenia** (days)	**1.09 (1.05–1.13)**	**<0.001**	**1.09 (1.04–1.13)**	**<0.001**
**Bacterial infection** (Yes vs. No)	**0.35 (0.16–0.76)**	**0.008**	**0.40 (0.18–0.88)**	**0.023**
**ECOG performance status** (0–1 vs. 2–4)	0.52 (0.16–1.69)	0.279	–	–
**ALL subtype** (B-ALL vs. T-ALL)	0.68 (0.26–1.77)	0.423	–	–

ALL: acute lymphoblastic leukemia; CI: confidence interval; ECOG: Eastern Cooperative Oncology Group; IFI: invasive fungal infection; OR: odds ratio; Ph: Philadelphia chromosome. A *p*-value < 0.05 was considered statistically significant. Variables with *p* < 0.10 in the univariate analysis were entered into the multivariate model. The final model was significant (χ^2^ = 22.3, *p* < 0.001), with a Nagelkerke R^2^ of 0.14, indicating moderate explanatory power. While the overall classification accuracy was 91.0%, the sensitivity for IFI-positive patients remained low (3.3%).

## Data Availability

The data supporting the findings of this study are available from the corresponding author upon reasonable request.
